# Pathophysiology in Mountain Yellow-Legged Frogs (*Rana muscosa*) during a Chytridiomycosis Outbreak

**DOI:** 10.1371/journal.pone.0035374

**Published:** 2012-04-25

**Authors:** Jamie Voyles, Vance T. Vredenburg, Tate S. Tunstall, John M. Parker, Cheryl J. Briggs, Erica Bree Rosenblum

**Affiliations:** 1 Department of Environmental Science, Policy and Management, University of California, Berkeley, California United States of America; 2 Department of Biology, San Francisco State University, San Francisco, California United States of America; 3 Department of Integrative Biology, University of California, Berkeley, California United States of America; 4 Department of Ecology, Evolution and Marine Biology, University of California Santa Barbara, Santa Barbara, California United States of America; 5 Animal Care Facility, University of California San Francisco, San Francisco, California, California United States of America; 6 Department of Ecology, Evolution and Marine Biology, University of California Santa Barbara, Santa Barbara, California United States of America; Smithsonian's National Zoological Park, United States of America

## Abstract

The disease chytridiomycosis is responsible for declines and extirpations of amphibians worldwide. Chytridiomycosis is caused by a fungal pathogen (*Batrachochytrium dendrobatidis*) that infects amphibian skin. Although we have a basic understanding of the pathophysiology from laboratory experiments, many mechanistic details remain unresolved and it is unknown if disease development is similar in wild amphibian populations. To gain a better understanding of chytridiomycosis pathophysiology in wild amphibian populations, we collected blood biochemistry measurements during an outbreak in mountain yellow-legged frogs (*Rana muscosa*) in the Sierra Nevada Mountains of California. We found that pathogen load is associated with disruptions in fluid and electrolyte balance, yet is not associated with fluctuations acid-base balance. These findings enhance our knowledge of the pathophysiology of this disease and indicate that disease development is consistent across multiple species and in both laboratory and natural conditions. We recommend integrating an understanding of chytridiomycosis pathophysiology with mitigation practices to improve amphibian conservation.

## Introduction

Fungi have not traditionally been regarded as highly virulent pathogens in terrestrial vertebrates [1]–[2]. Yet the fungal pathogen *Batrachochytrium dendrobatidis* (*Bd*), which causes a skin disease known as chytridiomycosis, is implicated in global amphibian declines and extinctions [3]–[5]. Resolving the mechanisms of pathogenesis is an imperative step in understanding how *Bd* can cause devastating declines [6]. The pathogen was described over a decade ago [7] and it has taken many years to gain a basic understanding of the physiologic effects of chytridiomycosis.

Insights into the pathophysiology of chytridiomycosis have come from controlled laboratory infection experiments, but many of the mechanistic details of pathogenesis remain unresolved [8]. Recent studies indicate that normal skin functioning is impaired in frogs with *Bd* infections [9]–[10]. These pathophysiological changes are particularly problematic for infected hosts because the skin of healthy amphibians tightly regulates osmotic balance (i.e. regulation of electrolyte concentrations relative to water, [11]–[12]). In frogs with severe cases of chytridiomycosis, the breakdown of skin transport processes is associated with reductions in blood plasma electrolyte concentrations [9]. Specifically, hyponatremia (low sodium) and hypokalemia (low potassium) have been reported in amphibians that develop clinical chytridiomycosis [9], [13]–[14]. These conditions are thought to cause asystolic cardiac arrest and death [9]. However, other causes of cardiac abnormalities (such as hypoxia or low blood oxygen) have not been conclusively ruled out as contributing factors. Thus, the details of *Bd* pathogenesis require further investigation [8].

It is unknown if the effects observed in laboratory experiments accurately characterize disease development in natural populations because to date no study has provided physiological data from *Bd*-infected amphibians in the wild. One natural system has provided the opportunity to investigate the pathophysiology of chytridiomycosis in greater detail, the Sierra Nevada of California. The population-level dynamics of chytridiomycosis have been studied intensively in this system [15] and have added to our understanding of *Bd* in host species both in enzootic [16] and epizootic [17] disease states.

In this system, *Bd* was initially detected in a small number of individuals and then spread within and between populations in a wave-like pattern across the landscape, causing mass die-offs in mountain yellow-legged frogs (a species complex consisting of *Rana muscosa* and *Rana sierrae*, [16]–[17]). *Bd* was detected in populations using a non-invasive, quantitative diagnostic technique that allows an estimate of the infection intensity in individual hosts [18]–[19]. In individual frogs, disease development and mortality were linked with intensity of infection; the *Bd*-load (number of infectious zoospores) increased until the infection intensity exceeded a threshold value (∼10,000 zoospore equivalents, [17]). Mortality and rapid population decline consistently occurred beyond this fungal load level, which established the quantitative diagnostic of *Bd*-load as a reliable indicator of severe disease [17]. Similar patterns of disease spread have been documented in other regions including other frog and salamander populations [20]–[22], but few systems have been studied in such fine detail as in the Sierra Nevada.

The comprehensive and integrated approach to studying the host-pathogen dynamics in populations of mountain yellow-legged frogs provided an opportunity to assess the pathophysiological responses to infection during a chytridiomycosis outbreak. We collected morphological and blood biochemistry measurements during an outbreak in order to 1) better understand the pathophysiology of chytridiomycosis and 2) determine if the physiological effects observed in laboratory inoculation experiments are analogous to disease development in wild amphibians. With this information we can identify the best treatments regimes for intervention during a chytridiomycosis outbreak.

## Methods

### Ethics statement

Sequoia-Kings Canyon National Park, University of California, Berkeley, San Francisco State University, and University of California, Santa Barbara Institutional Animal Care and Use Committees approved the scientific research and ethics permits for this project.

### Study area for animal collections

**Figure 1:**
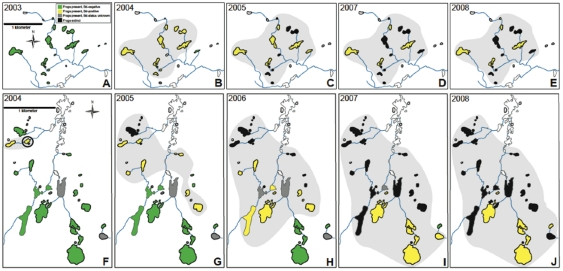
Maps of the two study basins where frog populations were monitored during chytridiomycosis outbreaks. Sequence of maps shows the spread of *Batrachochytrium dendrobatidis* (*Bd*) through Milestone Basin (A–E) and Sixty Lakes Basin (F–J). Lake color (green for *Bd*-negative populations, yellow for *Bd*-infected populations, and black for extinct frog populations) shows *Bd* infection and frog population status, and the light gray shaded region surrounds the area where frog populations were *Bd*-positive in each year. Open circle indicates the pond where *Bd* was first detected and where moribund frogs were collected for blood samples in 2004. Modified from [17].

Milestone Basin (36.647816° north, −118.454484°west; Figure 1 top row) and Sixty Lake Basin (37.180999°, −118.444582°; Figure 1 bottom row) are located in Sequoia–Kings Canyon National Park in subalpine-alpine zones (elevation 3,000–3,500 m). We sampled adult mountain yellow-legged frogs (*Rana muscosa*) on four occasions from July to October in 2004. Each sampling trip lasted approximately 3 weeks and during each trip we sampled 40–70 frogs at different lakes within the basins. We encountered frogs at the perimeters of water bodies in the lake basins and collected adult frogs with clean dip nets. We handled each individual using new latex gloves and recorded each animal's mass (measured to the nearest 0.01 g), snout-to-vent length (SVL).

### Diagnostics

We collected non-invasive skin swab samples from each individual by rubbing a sterile, synthetic-cotton swab over the ventral surfaces and digits [17], [19]. Skin swabs were air-dried in the field for five minutes and stored in a cool location until they could be transported to the laboratory. In the laboratory, we stored swabs 4°C until analysis, which occurred a four-month period. We used a quantitative measure of *Bd* genomic equivalents using a Taqman real-time polymerase chain reaction (PCR) assay [18]–[19]. We used a standard protocol [18] for our extraction methods and universal *Bd* standards (0.1–100 zoospore equivalents run in triplicate) provided by A. Hyatt.

### Blood biochemistry

We collected blood samples (∼5–10% of body weight) via cardiac puncture [12] using 50 cc syringes. We centrifuged blood samples (11,000 RPM, ZipoCrit centrifuge) immediately in the field and used an i-STAT portable clinical analyzer (Heska Corporation, Loveland, CO, USA) to assess blood parameters in the field. In some cases the limited blood volume (e.g. from sub-adult frogs) restricted the number of analyses that could be accurately performed. In those cases, we omitted unreliable results from our analysis. We used the following plasma biochemical parameters for statistical analysis: hematocrit, total protein, blood urea nitrogen content, pH, total carbon dioxide, partial pressure carbon dioxide, partial pressure oxygen, bicarbonate, base excess extracellular fluid, anion gap, glucose, sodium, potassium and chloride.

### Statistics

We analyzed the data with R, version 2.7.1 (www.r-project.org). We calculated the body condition index (BCI) by taking the residuals of the linear regression of body mass on snout-to-vent length [23]. We tested for normality (Q-Q plots and Shapiro-Wilk's test) and used a log transformation where violations of normality assumptions occurred.

Due to the high number of dependent variables, we first analyzed for dimension reduction with Principle Components Analysis (PCA) using the NIPALS algorithm [24]. We used Cattell's scree test to decide number of components and included eigan values >1 [25]. We used factors with loadings of 0.3 or greater for interpretation and multiple regression analysis to determine if relationships existed between *Bd*-load (genetic equivalents with a log transformation, log10 (n+1)) and the principle components. We then ran correlation tests on parameters that loaded highly (>0.3) on principle components that were significantly associated with *Bd*-load. Lastly, we grouped amphibians into categories of uninfected, low (1–10,000) and high (over 10,000) zoospore equivalents to determine if there were clear differences among groups of amphibians based on the 10,000 zoospore threshold value that is thought to be critical for amphibian mortality in these species [17].

## Results

**Figure 2:**
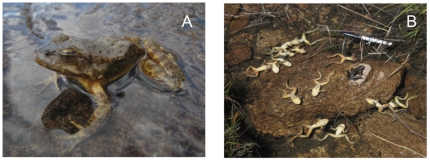
Appearance and behavior of mountain yellow-legged frogs (*Rana muscosa*) and during a chytridiomycosis outbreak in Sixty Lakes Basin, Sierra Nevada Mountains, California. A) A frog showing clinical signs of severe chytridiomycosis including abnormal posture. B) Dead frogs following a chytridiomycosis outbreak in Milestone Basin.

We detected *Bd* from diagnostic skin swabs in Milestone Basin in July 2004. By October of 2004, *Bd* had spread throughout the entire basin (Figure 1) and frogs showed clinical signs of chytridiomycosis (Figure 2). Over the course of four sampling trips, we collected and analyzed blood biochemistry measurements from adult frogs (*n* = 121).

Two principle components accounted for 55% of the variance in the data and were selected based on the criteria described above. A pattern matrix (Table 1) shows the component loadings for each factor including BCI and blood biochemical parameters. PCA analysis indicated that discrete biochemical parameters could be grouped to represent more general physiological factors. Factor names for each of the two principle components (“Acid-base balance” and “Fluid and electrolyte balance”) were selected based on the parameters with high loadings (>0.3) and on general knowledge of biological functions of each parameter.

**Table 1:** Component loadings of blood biochemistry parameters measured in mountain yellow-legged frogs infected with *Batrachochytrium dendrobatidis*.

Variable	PC1	PC2
	*Acid-base balance*	*Fluid and electrolyte balance*
Body Condition Index	0.06	**0.34**
Hematocrit	0.01	−0.24
Protein	0.08	**−0.34**
Blood Urea Nitrogen	−0.01	−0.29
pH	**0.45**	−0.01
Total Carbon Dioxide	**0.52**	0.04
Partial Carbon Dioxide	0.28	0.16
Partial Oxygen	−0.02	0.02
Bicarbonate	**0.39**	−0.05
Base Excess Ext. Fluid	**0.54**	0.00
Anion Gap	0.00	−0.09
Glucose	0.03	**−0.48**
Sodium	0.02	**0.38**
Potassium	−0.03	−0.29
Chloride	−0.02	**0.36**
Eigenvalue	1.60	1.23

Interpretable factor loadings (>0.3) are in boldface.

Acid-base balance is the maintenance of proper pH levels in the blood [26]. Parameters that loaded highly (>0.3) for acid-base balance included pH, total carbon dioxide, bicarbonate and base excess extracellular fluid. When assessed for a relationship with *Bd*-load, there was no statistically significant correlation (Pearson standard parametric correlation: r = −0.12, df = 119, *P*-value = 0.20).

**Figure 3:**
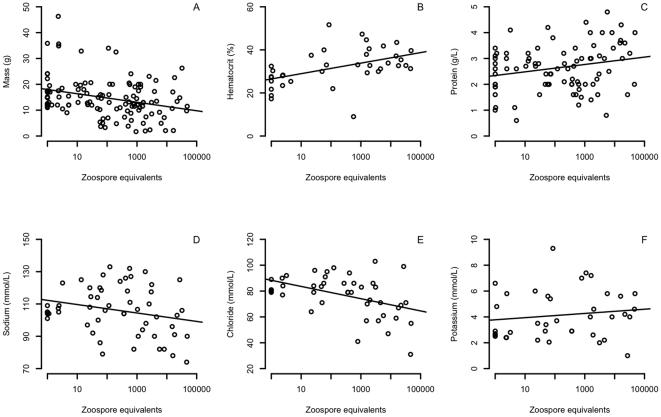
Mass and blood biochemistry parameters collected from mountain yellow-legged frogs during a chytridiomycosis outbreak. A–C) Mass, hematocrit and protein values suggest dehydration in frogs with high loads of *Batrachochytrium dendrobatidis* (*Bd*). D–F) Plasma electrolyte concentrations (sodium and chloride) indicate abnormalities in fluid and electrolyte balance in frogs with high *Bd*-loads.

Fluid and electrolyte balance is characterized by the correct ratios of solutes to fluids (i.e. water) and generally indicates osmotic equilibrium or hydration status. Parameters that loaded highly (>0.3) for fluid and electrolyte balance included BCI, protein, glucose, sodium, potassium, and chloride. This principle component was significantly correlated with *Bd*-load (Pearson standard parametric correlation: r = 0.28, df = 119, *P*-value = 0.002). Of the parameters that loaded highly on the fluid and electrolyte balance principle component, all except BCI and plasma potassium concentrations were significantly correlated with *Bd*-load (Table 2). We also assessed hematocrit and mass as individual parameters because they are strong indicators of hydration status. We found positive correlations between *Bd*-load and hematocrit (Pearson standard parametric correlation: r = 0.54, df = 32, *P*-value<0.001; Figure 3B), and a negative correlation between *Bd*-load and mass (Pearson standard parametric correlation: r = −0.25, df = 98, *P*-value = 0.009; Figure 3A).

**Table 2:** Pearson correlation between *Bd*-load (genomic equivalents determined by qPCR analysis) and parameters that loaded highly on the Fluid and electrolyte balance component.

Parameter	r	df	*P*-value
Body Condition Index	−0.02	119	0.75
Protein	0.3	73	0.02*
Glucose	0.37	66	0.001*
Sodium	−0.24	61	0.05*
Potassium	0.03	45	0.79
Chloride	−0.44	42	0.002*

**Figure 4:**
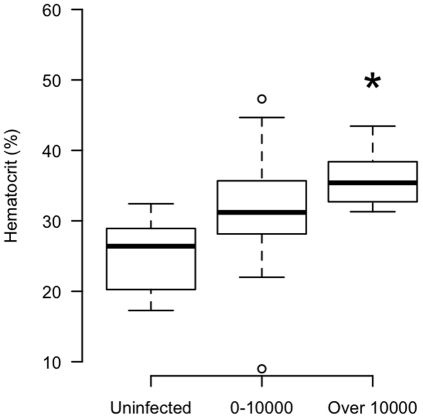
Hematocrit in yellow-legged frogs grouped by intensity of infection with *Batrachochytrium dendrobatidis*. The group of frogs with over 10,000 zoospore equivalents had significantly different hematocrit percentages compared with the group that was uninfected (Tukey HSD, *P* = 0.02).

When also grouped amphibians into categories of uninfected, low (1–10,000 zoospores) and high (over 10,000 zoospores) infection intensities, our results suggested possible differences among groups for each physiological parameter. However, with the exception of hematocrit (ANOVA, *P*<0.01, Tukey HSD, *P = *0.02 for control and over 10,000 zoospore equivalent groups; Figure 4), these differences were not significant, probably due to the variability among individuals.

## Discussion

The well-studied die-offs in mountain yellow-legged frogs (*Rana muscosa*) have characterized the nature of chytridiomycosis-related amphibian declines occurring around the world; *Bd* spreads rapidly into naïve amphibian populations and causes high levels of mortality. The frog populations of the Sierra Nevada were screened for *Bd*-infection from 1996–2008 and, following the initial detection of *Bd* in 2004, were reduced by >75% (Figure 1, [17]). Quantification of *Bd*-load over a relatively short sampling period (months before and after the detection of *Bd*) demonstrated a rapid increase in infection intensity in the dead and dying individuals from these populations. Thus, mortality in these natural populations was inextricably linked with the spread of *Bd* and the subsequent increase in infection intensities [17]. However, no mechanistic explanation for mortality in wild amphibian die-offs was previously available. Our study provides new pathophysiological data collected from natural populations during a chytridiomycosis outbreak and adds new information to understand the mechanisms of mortality in chytridiomycosis. Based on these results, we suggest a more informed approach to treating diseased amphibians during chytridiomycosis outbreaks.

Our results indicate that increases *Bd*-load, which were documented in great detail during this outbreak, were significantly associated with indicators of fluid and electrolyte balance. Specifically, we found that increasing *Bd*-load is associated reductions in plasma electrolyte concentrations, and we also found some evidence of dehydration. Taken together, these two findings suggest that frogs with severe chytridiomycosis have a greater loss of solutes from circulation than could be determined with electrolyte concentration measurements alone.

Even moderate dehydration with concomitant reductions in solute concentrations suggests that frogs with chytridiomycosis have a more pronounced loss of electrolytes than previously estimated. Fluid and electrolyte balance is normally evaluated by measures of blood solute concentrations relative to body condition index (BCI), which is determined by measures of body mass relative to body size (snout-to-vent length, SVL). An increase in blood solute concentrations, and especially in hematocrit, accompanied by a decrease in body mass would point toward dehydration. In contrast, over-hydration is indicated by a decrease in blood solute concentrations with an increase in body mass. We found negative correlations between *Bd*-load and body mass, and positive correlations between *Bd*-load and protein and hematocrit concentrations (Figure 3 A, B, C). However, there were negative relationships between *Bd*-load and other solute concentrations, such as sodium and chloride (Figure 3 D, E, Table 2). Other laboratory studies have shown similar dramatic decreases in electrolyte concentrations, but with only weak evidence of dehydration [9], [13]–[14]. However, these studies are not directly comparable because the environment in the laboratory does not simulate natural conditions, sample sizes were considerably smaller compared to the present study and, in some laboratory tests, electrolyte concentrations were below the detection limit of the available equipment [9]. Thus, our results support the hypothesis that fluid and electrolyte imbalance is a critical component of the pathophysiology of chytridiomycosis, but they also suggest that electrolyte loss is even greater than previously thought in diseased frogs.

Our results also indicate that acid-base balance is not considerably altered in amphibians with severe chytridiomycosis. Acid-base balance, which requires multi-system regulation [26], is one facet of *Bd* pathophysiology that has been difficult to study. Blood pH levels are determined metabolic (e.g. bicarbonate generation in the kidney, known as the bicarbonate buffering system) and respiratory (e.g. carbon dioxide production in various tissues) mechanisms, both of which act in concert to correct for perturbations in pH that result in acidosis or alkalosis [26]. Carver et al. [10] tested oxygen consumption and carbon dioxide production using flow-through respirometry; they found no evidence of altered metabolic rates in infected frogs. Voyles et al. [14] measured partial pressure carbon dioxide in blood samples and oxygen saturation (using pulse oximeter; [9]) and did not find significant changes in respiratory gases in severely diseased frogs. However, these previous studies were limited in their ability to assess blood pH and bicarbonate. Our study provides information on these parameters (Table 1), and suggests there is little evidence of changes in acid-base balance in diseased frogs.

Electrolyte depletion has been the most consistent finding in studies that have aimed to determine the physiological effects of chytridiomycosis, and our study documents these pathophysiological changes in free-living amphibian populations during an outbreak. These results indicate that pathophysiological responses to an increase in *Bd*-load in amphibian epidermis are consistent across multiple species and between captive and wild amphibian populations. Therefore, optimized treatments that correct pathophysiological abnormalities could be invaluable where wild amphibian populations experience chytridiomycosis outbreaks and *Bd*-induced declines.

Treatment regimes that aim to decrease fungal load in wild amphibian populations may be the best strategy to mitigate outbreaks of chytridiomycosis [27], and the success of field treatments will be considerably enhanced by a detailed understanding of disease development and pathogenesis. Many different strategies to reduce fungal loads of amphibians in a natural setting have been suggested [28], but few have been attempted during chytridiomycosis outbreaks in the wild. One approach that holds promise is treating individual amphibian hosts with antifungal solutions in the wild (e.g. using fungicidal chemicals or anti-*Bd* “probiotic” treatments, [28]), which is now being trialed for *Rana muscosa* and *Rana sierrae* (V. Vredenburg, *unpublished work*).

The success of implementing treatment strategies in wild amphibians will hinge on the timing of treatment, and also on the effectiveness of treatments in reducing *Bd*-load and correcting pathophysiological abnormalities. The timing of treatments is important because the damage may be irreparable in the final stages of disease progression. The effectiveness of anti-*Bd* agents is critical to reduce *Bd*-loads and suppress the advancement of disease. We suggest that electrolyte supplementation should be used in conjunction with antifungal treatments to reduce the risk of mortality during outbreaks when frogs are showing clinical signs of disease. Berger et al. [27] reviewed multiple clinical trials designed to optimize the use of both antifungal treatments and isotonic electrolyte supplementation. This approach has been attempted with some success in clinical settings (S. Young, *unpublished work*). The optimal combination for a particular species may require additional background work to determine the best treatments accounting for host life-stage, behavioral and/or ecological characteristics [27]. Nonetheless, the application of well-informed treatment practices in wild amphibian populations is an attainable goal for amphibian conservation biologists. Treatment regimes for individual hosts that incorporate an understanding disease progression may prove to be an indispensible tool for mitigating chytridiomycosis outbreaks, facilitating population recovery and confronting the conservation challenges associated with chytridiomycosis and amphibian loss.
